# Peutz-Jeghers syndrome: A case series

**DOI:** 10.1016/j.ijscr.2024.110117

**Published:** 2024-08-03

**Authors:** Narendra Pandit, Durga Neupane, Kunal Bikram Deo

**Affiliations:** a Department of Surgical Gastroenterology, Birat Medical College Teaching Hospital, Morang, Nepal; b Department of Surgical Gastroenterology, BP Koirala Institute of Health Sciences, Dharan, Nepal

**Keywords:** Peutz-Jeghers syndrome, Polyps, Hamartomatous polyp, Resection, cancer, Case series

## Abstract

**Introduction:**

Peutz-Jeghers syndrome (PJS) is a rare hereditary disorder characterized by gastrointestinal hamartomatous polyps, due to mutation of the STK11/LKB1 gene located on chromosome 19p. The polyps are most commonly found in the small bowel followed by colon.

**Case presentation:**

Our case series includes 4 patients, three being male and one female. Each of them either presented with abdominal pain and other associated symptoms. Oral cavity and lip melanin pigmentation were common. CT abdomen revealed multiple large jejunal, ileal, gastric and colon polyps. Cancer was found in one patient. Different surgical approaches were adopted. All recovered well.

**Discussion:**

PJS is an autosomal dominant disorder with an estimated incidence of 1:50,000 to 1:200,000 cases with a significant family history. Mostly found in small bowel followed by colon, it can also occur in a rare organ like gall bladder as evident in our case. PJS carries a substantial risk for gastrointestinal cancer. The treatment modality depends on the site of polyp, mode of presentation and availability of the expertise.

**Conclusion:**

PJS is a common disease in our part which is usually observed in teen age groups male. They have a varied presentation, from intestinal obstruction (due to intussusception) to GI bleeding. Colonic malignancy at young age may be the first presentation of the disease. Observation of melanin pigmentations on lips helps diagnose the disease; and one should always look at this findings in a young patient with pain abdomen or in intestinal obstruction to confirm/exclude the disease.

## Highlights

• Peutz-Jeghers syndrome (PJS) presents with gastrointestinal hamartomatous polyps.
• The polyps are most commonly found in the small bowel followed by colon and rarely gallbladder.
• PJS is a common disease in our part which is usually observed in teen age groups male.
• They have a varied presentation, from intestinal obstruction (due to intussusception) to GI bleeding.
• Colonic malignancy at young age may be the first presentation of the disease.

## 1 Introduction

Peutz-Jeghers syndrome (PJS) is a rare hereditary disorder characterized by gastrointestinal (GI) hamartomatous polyps, due to mutation of the STK11/LKB1 gene located on chromosome 19p. Approximately half of the cases are inherited, while the remaining are sporadic as a result of spontaneous mutation [1,2]. The polyps are most commonly found in the jejunum (90 %), followed by ileum (80 %), colorectal (50 %) and stomach (25 %). They have an estimated 15-fold increased risk of developing intestinal and extra-intestinal cancer compared to the general population. Around 50 % of patients with PJS develop and die from cancer by the age of 57 years; and the mean age at first diagnosis of cancer is 43 years [[3], [4], [5]].

Juvenile polyposis syndrome and Peutz-Jeghers syndrome are hereditary polyposis syndromes characterized by hamartomatous polyposis in the gastrointestinal tract and increased risk of cancer. These diagnoses are based on clinical criteria, most often supported by the detection of germline pathogenic loss-of-function variants in STK11, BMPR1A, or SMAD4 [6].

PJS onset may occur at any age and the risks of gastrointestinal and extra-gastrointestinal cancer are high. Thorough surveillance of PJS patients for malignancies is vital [7].

Studies reporting the development of malignancies in hamartomatous polyps support the hamartoma-adenoma-carcinoma sequence hypothesis. However, since the malignant potential of PJS polyps has not been established, it is not clear whether endoscopic polypectomy can prevent cancer or reduce cancer risk [8].

Here, we review our experience in management of series of four PJS patients over a period of 11 years. Our case series has been reported in line with the PROCESS guideline [9]. Table 1 shows clinical characteristics of patients with PJS.

**Table 1:** Clinical characteristics of patients with PJS.

S.N	Age (yr)/sex	Presentation	Location of polyp	Malignancy	Family history
1	9/M	Intussusception	Jejunum	No	Yes
2	16/M	Intussusception	Ileum, cecum	No	Yes
3	22/F	Hematochezia	Jejunum, transverse colon, gallbladder	No	No
4	18/M	Acute Intestinal obstruction (colonic growth)	Jejunum, ileum, right colon	Yes (transverse colon, stage III)	No

## 2 Case presentation

On chronological order of management:

The *first case* was a 9-year-old male who presented to the emergency department with abdominal pain for 1 day. It was sudden onset, severe, and progressive associated with vomiting and obstipation. Abdominal examination revealed generalised tenderness with guarding and rigidity. X-ray abdomen showed multiple air-fluid level. Blood investigation were normal apart from leucocytosis. The patient was resuscitated, injectable broad spectrum antibiotic initiated, and proceeded for emergency laparotomy. At surgery, there was a long segment jejuno-jejunal intussusception, which was reduced. On reduction, the 10 cm segment of jejunum was gangrenous, which was resected and anastomosed. The lead point was a jejunal polyp of size 4 cm. There was another polyp (2 cm), 10 cm proximal to the gangrenous segment, which was removed by enterotomy. On inquiry, the father gives history of a family history of gastrointestinal (GI) polyposis which included his three generations. There was also the presence of mucocutaneous melanin pigment in the oral cavity and lips. The patient did well, discharged on Day 10. At 11 years follow-up, there is no recurrence of symptoms.

The *second case* was an elder brother (16-year) of the above patient in the same family tree. He presented with complaints of recurrent abdominal pain for last 1 year. It was associated with loose stool- on/off. The patient denied any history of bleeding per anum, vomiting, or hospitalization. On examination, there was a tenderness in the right iliac fossa. The patient also had an oral cavity and lip melanin pigmentation. On imaging with contrast computed tomography (CT), there was an evidence of ileocolic intussusception. The apex of the intussusception contained a well-defined soft tissue density of size 5 cm. The patient was taken up for surgery. At surgery, there was an ileocolic intussusception, reaching up to the ascending colon, which was irreducible. Hence, limited right hemicolectomy with ileocolic anastomosis was done. On cut-section, there was a pedunculated polyp in the cecum (4 cm), and another polyp, 10 cm proximal to ileocecal junction, leading to intussusception. The histopathology of both patients confirmed hamartomatous polyp. This patient also had no recurrence of symptoms at seven years of follow-up.

**Fig. 1:**
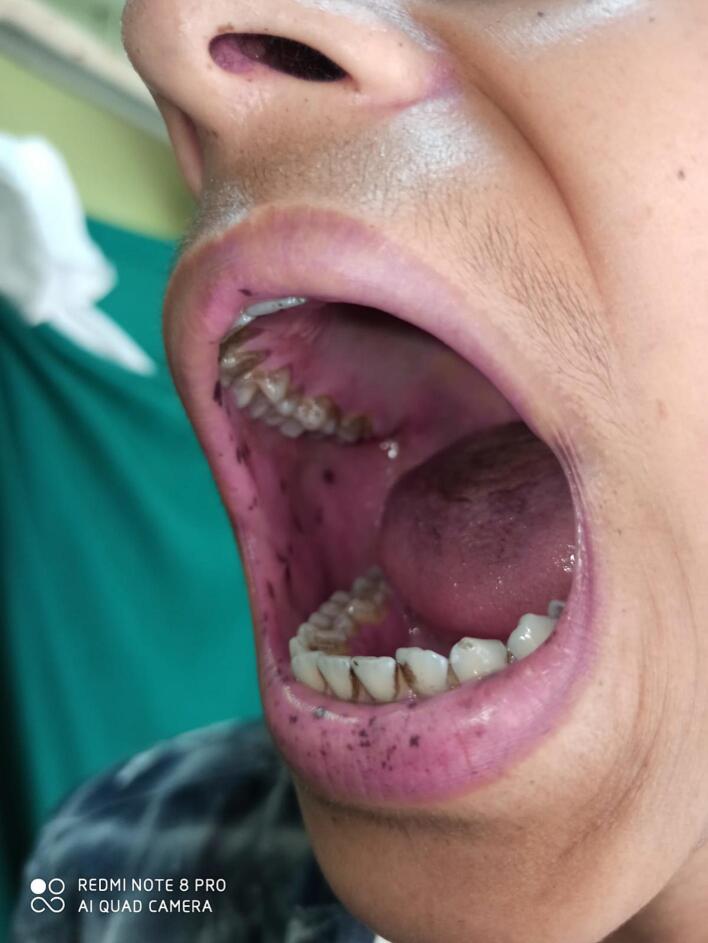
Pictorial image showing characteristic melanin pigmentation on lips and buccal mucosa.

**Fig. 2:**
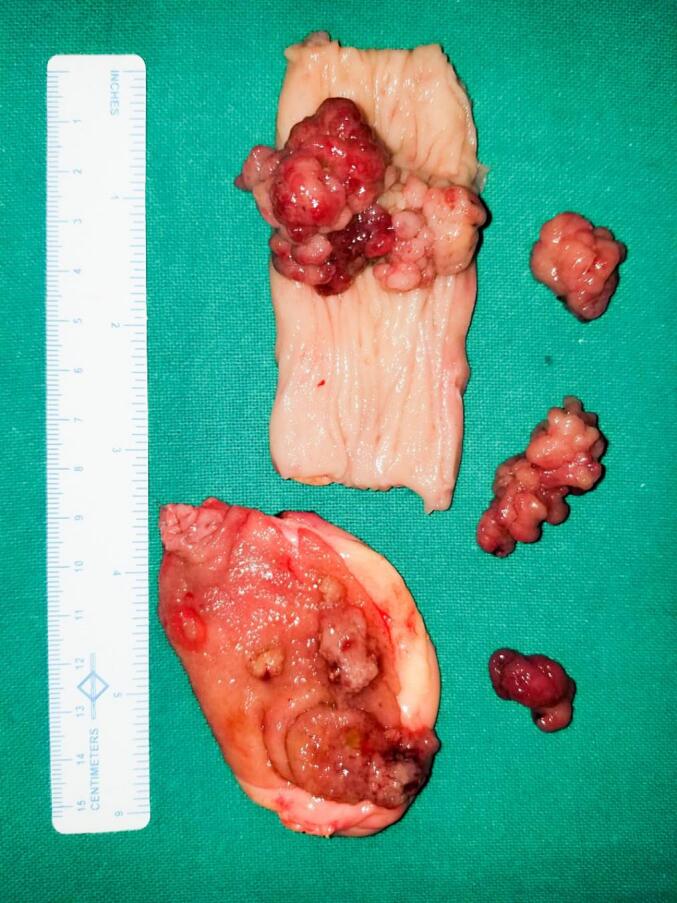
Operative image showing gallbladder polyp, transverse colon polyp, and multiple excised jejunal polyps.

**Fig. 3:**
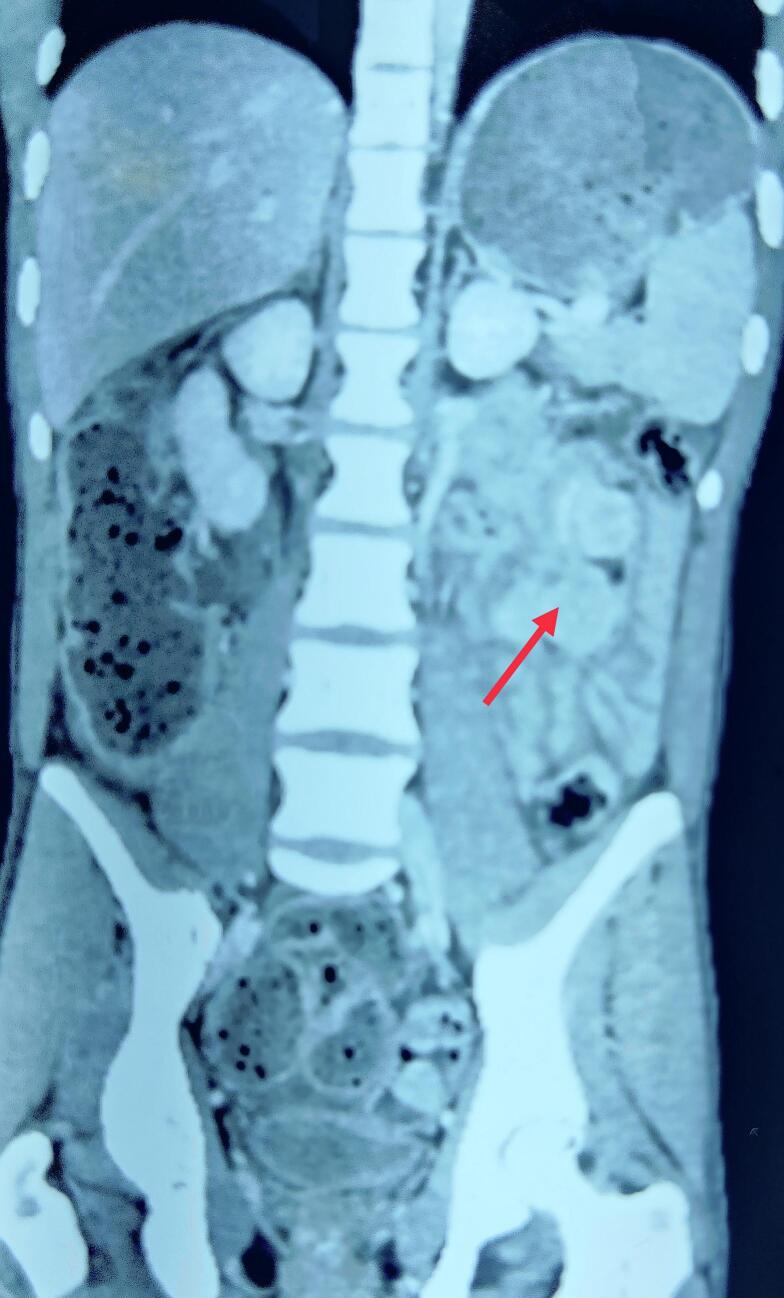
CT coronal image shows multiple jejunal polyps (arrow).

The *third case* was a 22-year-old female, who presented with lower GI bleed for last 8 months. It was intermittent, fresh, without history of blood transfusion. She also had evidence of dark, mucocutanoeus melanin pigmentation of the lip (Fig. 1). She denied any family history of GI polyposis. The blood investigations were normal including haemoglobin. On contrast CT abdomen, there was transverse colon polyps (3 cm size, two in number), gallbladder polyp (3 cm), and jejunal polyps. There was no evidence of intussusception. She underwent segmental resection (6 cm) and anastomosis of the transverse colon containing polyps, cholecystectomy and enterotomy of the jejunum at three sites and polypectomy as part of *clean sweep* procedure (Fig. 2). Fig. 3 shows the CT finding with multiple jejunal polyps. She did well postoperatively. The biopsy confirmed hamartomatous polyp. The gallbladder polyp was too non-malignant. At 4 years follow-up, she is asymptomatic.

**Fig. 4:**
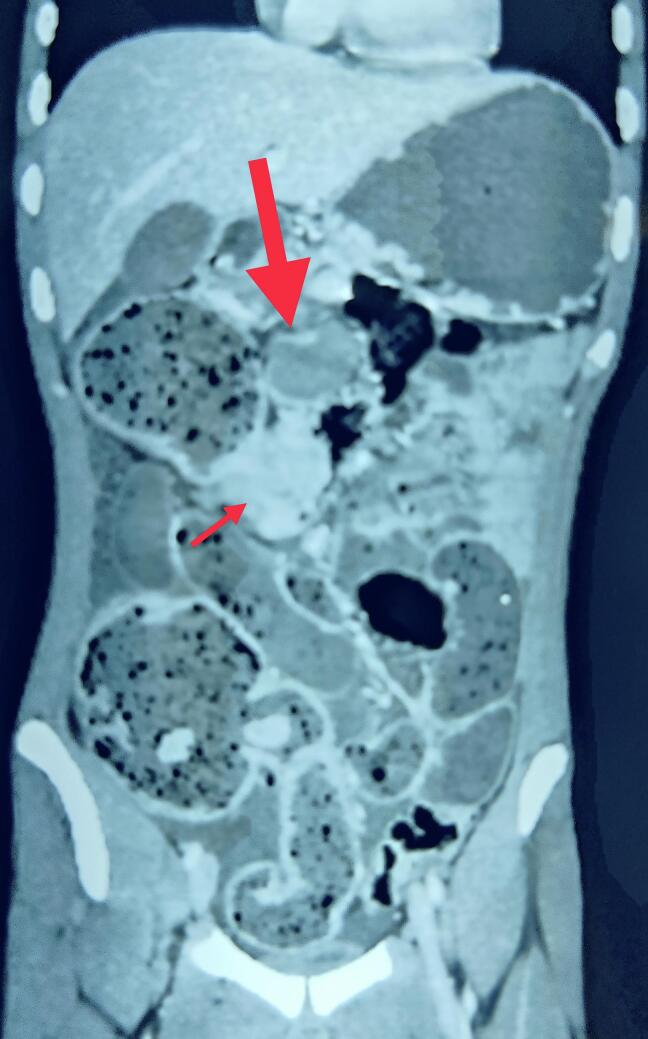
Large metastatic node (thick arrow) Transverse colon shows irregular, heterogenous mass with a prominent “small bowel faeces sign” (thin arrow).

**Fig. 5:**
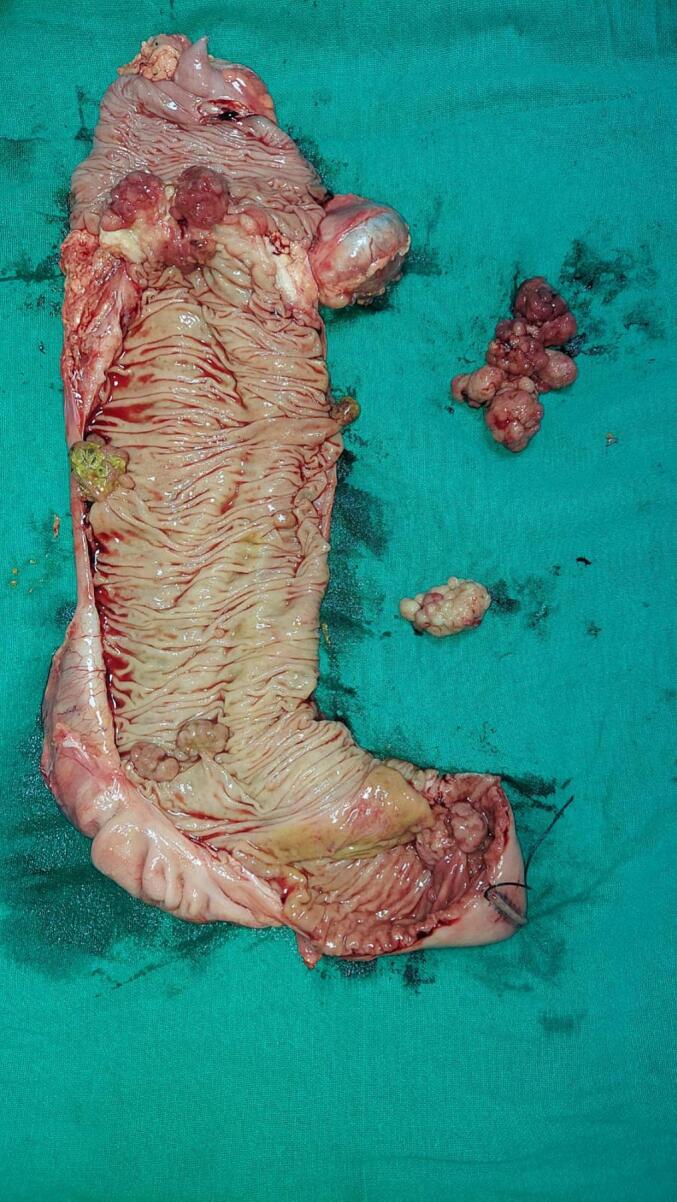
Right hemicolectomy specimen showing transverse colon mesentery, multiple colonic polyps, and resected jejunal polyp on green sheet. (For interpretation of the references to colour in this figure legend, the reader is referred to the web version of this article.)

The *fourth patient* was 18-year-old male who presented to the emergency with features of acute intestinal obstruction of a week duration (abdominal distention, obstipation and vomiting). There was no history of prior hospitalization, bleeding or pain abdomen. He denied any family history of GI polyposis or any illness. On examination, there was melanin pigmentation on the lips, abdomen distended but non-tender. Abdominal x-ray showed multiple air-fluid level. The contrast CT abdomen revealed multiple large jejunal, ileal, gastric and colon polyps. There was a transverse colon mass lesion, which was 5 cm size, and was the cause of obstruction. Further, there was a significant, large (5 cm), node at the colonic mesentery. There was also the presence of small bowel faeces sign suggesting prolonged intestinal obstruction (Fig. 4). Blood investigation showed anaemia (Haemoglobin- 8 g/dl and albumin- 2.5 g/dl) and hypoalbumineamia. He underwent emergency surgery with right hemicolectomy, complete mesocolic resection, end ileostomy, proximal jejunal enterotomy (at two sites), and polypectomy. Cut section of the specimen revealed transverse colon annular growth, multiple ascending colon and terminal ileal polyps (Fig. 5). The pathology report confirmed moderately differentiated adenocarcinoma of colon with involvement of 10 mesocolic lymph nodes (pathological stage III disease). Postoperative, he underwent adjuvant chemotherapy. At three months follow-up, he is doing well.

## 3 Discussion

PJS is an autosomal dominant disorder with an estimated incidence of 1:50,000 to 1:200000 cases [10]. Around 50 % of cases have family history suggestive of PJS, while the remaining may be a new cases. Majority are diagnosed in teen ages as seen in the present series. The polyp can occur anywhere in the GI tract, but the most common site is small bowel followed by the colon [1]. Surprisingly, the polyp may be observed in the gallbladder as was observed in one of our female patient. They usually present with symptoms of intestinal obstruction due to the polyp. The polyp acts as a lead point causing intussusception. However, the obstruction may be due to the *de novo* colonic malignancy [4,11]. It may be the first presentation as was observed in one of our patient.

The second common presentation is bleeding from the polyp. The bleeding is due to the auto-amputation of the polyp leading to polyp site mucosal bleed. This leads to anaemia, and multiple blood transfusion [1,12]. As per the WHO criteria, diagnosis is established by the presence of two or more histologically confirmed hamartomatous GI polyp; any number of polyp in a patients with family history of PJS; or presence of any GI polyp with mucocutaneous melanosis at vermillion border of lip, buccal mucosa, palm or anal margin; or characteristic mucocutaneous pigmentation in an individual who has a family history of PJS [2].

These patients are at life time risk of multiple malignancy (50 %). The common malignancy observed in these patients are colon cancer, small intestinal cancer, pancreatic cancer, thyroid cancer, and breast or testis cancer. The malignancy rarely develops in the hamartomatous polyp, rather these patients are associated with development of malignancies *de novo*. Their life-span is short; hence requires regular surveillance of development of any malignancy [4,5]. Treatment of polyp is by “clean sweep” polypectomy either by double-balloon enteroscopy, surgical enterotomy and on table endoscopy, or by segmental resection for heavily involved small bowel segment. The treatment modality depends on the site of polyp, mode of presentation and availability of the expertise [1,13,14]. Our three patients, who are in follow-up for eight, six and five years are doing well, without any malignancy. The last patient with colonic cancer is receiving adjuvant therapy for colonic cancer.

## 4 Conclusion

PJS is not an uncommon disease in our part. It is usually observed in teen age groups male. They have a varied presentation, from intestinal obstruction (due to intussusception) to GI bleeding. Colonic malignancy at young age may be the first presentation of the disease. Observation of melanin pigmentations on lips helps diagnose the disease; and one should always look at this findings in a young patient with pain abdomen or in intestinal obstruction to confirm/exclude the disease.

## Consent

Written informed consent was obtained from the patient for publication of this case series and accompanying images. A copy of the written consent is available for review by the Editor-in-Chief of this journal on request.

Written informed consent was obtained from the patients' parents for publication of this case series and accompanying images. A copy of the written consent is available for review by the Editor-in-Chief of this journal on request.

## Ethical approval

Ethical clearance for case reports and case series is not required according to IRB of our institution.

## Funding

None.

## Author contribution

All the authors contributed equally in preparation of this manuscript. Final version is approved by all authors.

Narendra Pandit: Conceptualization; Methodology; Investigation; Writing-Original Draft; Writing -Review and Editing; Visualization; Supervision; Project Administration.

Durga Neupane: Conceptualization; Methodology; Investigation; Writing-Original Draft; Writing -Review and Editing; Visualization; Supervision; Project Administration.

Kunal Bikram Deo: Conceptualization; Methodology; Investigation; Writing-Original Draft; Writing -Review and Editing; Visualization; Supervision; Project Administration.

## Guarantor

Narendra Pandit.

## Research registration number

N/A.

## Conflict of interest statement

None.
